# Role of calcium carbonate in the process of heavy metal biosorption from solutions: synergy of metal removal mechanisms

**DOI:** 10.1038/s41598-022-22603-4

**Published:** 2022-10-21

**Authors:** Sławomir Wierzba, Joanna Makuchowska-Fryc, Andrzej Kłos, Zbigniew Ziembik, Wioletta Ochędzan-Siodłak

**Affiliations:** 1 Institute of Environmental Engineering and Biotechnology, University of Opole, Kard. Kominka 6a, 45-040 Opole, Poland; 2 Faculty of Chemistry, University of Opole, Oleska 48, 45-052 Opole, Poland

**Keywords:** Environmental biotechnology, Pollution remediation, Chemical engineering

## Abstract

The effect of calcium carbonate on the removal efficiency of cations of the selected heavy metals Cu, Zn and Pb from aqueous solutions using various biosorbents (BS) was investigated under laboratory static conditions. The main mechanism of biosorption of heavy metal cations is ion exchange, whereas the reaction with calcium carbonate results in precipitation of poorly soluble carbonates and hydroxides of the examined heavy metals. Studies conducted under static conditions have shown that the effect of Cu and Zn cations removal from solutions is better when using a mixture of BS and CaCO_3_ as compared to the effect of process, in which these two components were used separately. Removal efficiency for Cu and Zn has been shown to increase from 20 to 50% depending on the BS used. For the removal of lead cations, a measurable effect is found only for biosorbents whose active centers are saturated with protons (improvement in removal efficiency by about 20%). A synergy effect in the flow system was also investigated. It was found that under the conditions of the experiment, the addition of powdered CaCO_3_, in a weight ratio of 1 g CaCO_3_: 15 g BS, increases the removal efficiency of all the metals studied by 20–30%. It has been shown that an important role in the process of heterophasic ion exchange is played by neutralization of protons—desorbed from the biosorbents—with hydroxide ions released into the solution by partial dissolution of CaCO_3_ and subsequent hydrolysis reaction.

## Introduction

Surface water pollution, including heavy metals, is a serious environmental problem of global importance. Untreated wastewater discharge leads to an increasing level of pollution of the seas and oceans, which is also caused by contaminated rainwater and drainage from fields. Global water pollution is evidenced by the increasing level of pollution in the Arctic waters. It has been found, for instance, that one of the sources of Pb and Hg origin in Svalbard is a sea aerosol^1^. Also, the presence of other anthropogenic elements is recorded in the Arctic waters, as exemplified by the results of tests on the presence of heavy metals accumulated in narwhal organisms^2^. In this aspect, an important element of water quality protection is the elimination of threats at source—even during the stage of wastewater generation. In order to remove heavy metal cations from water, many conventional treatment techniques are used, such as chemical precipitation, ion exchange, electrochemical treatment, membrane technologies, adsorption, etc.^3^, and for several decades research has also focused on the possibilities of using biosorbents, often constituting waste products. It is believed that the removal of metal cations from waters by means of biomass is a cheap process and is characterized by high efficiency^4,5^. Large amounts of waste biomass with good sorption properties are provided for instance by agriculture, forestry and the agri-food industry. For removal of heavy metals from aqueous solutions, among others, cellulose^6^, saw dust^7,8^, bark^9^, straw^10^, nut shells^11,12^ cereal bran^13,14^ and other materials are used. The processes of biosorption have also been intensified by means of various types of modifications, for example preparation of biosorbent mixtures^15^ or by methods requiring conversion of biomass into biochar^16^.

Another way of removing heavy metals from solutions is to create poorly soluble metal compounds in the form of hydroxides or carbonates. For this purpose, mineral calcium carbonate and its biological forms, e.g. waste shells of chicken eggs, are used. Numerous studies describe the mechanisms that are prevalent in the process of removal of heavy metals from solutions depending on the nature of carbonates, which can be seen in the different structure of materials^17–19^. Calcium carbonate of geological origin has low efficiency in heavy metal adsorption^20^, and the main removal mechanism is the exchange reaction with the formation, as mentioned, of poorly soluble metal compounds. Therefore, most studies use carbonates of biological origin: shells of chicken eggs, or shells of clams or snails^21–25^. Many works concern the increase in the contact of surface of carbonates of various origins, by means of their chemica^26^, thermal^27^ or mechanical modification^28^.

There are a few studies, in which calcium carbonate was used in combination with other treatment technologies, for instance the use of calcined chicken egg shells and microalgae^29^, the combination of heavy metal precipitation using chicken egg shells with a membrane biological reactor^30^, or the use of calcium carbonate as a precipitation additive in the process of heavy metal wastewater treatment with biopolymers^31^. Methods of modification of biosorbents with calcium carbonate are also known. Biochars, often used for wastewater treatment, are modified with nano-scale materials, e.g. calcite, vaterite, amorphous calcium carbonate or their hybrids. Biochar—prepared from sewage sludge—with the surface modified by means of a synthetic calcite, was also used for removing cadmium from solutions^20^. The efficiency of lead and cadmium biosorption using functionalized yeast cells with intracellular CaCO_3_ mineral reinforcement (skeleton)^32^, as well as biosorption on immobilized bacterial cells in various composites, for instance those made from alginate, polyvinyl alcohol and CaCO_3_^33^, was also studied.

To date, however, no studies have been undertaken on the synergistic interactions taking place during the following processes: heterophasic ion exchange in biosorbent—solution and chemical exchange reaction with calcium carbonate, leading to precipitation of heavy metals in the form of poorly soluble carbonates and hydroxides. These processes have been successfully applied in the already mentioned system of coarse precipitation of heavy metals with calcium carbonate from mine drainage and subsequent purification in a reactor with microalgae^29^.

The aim of the presented studies was to evaluate the removal efficiency of heavy metal cations: Cu, Zn and Pb from aqueous solutions while using various biosorbents and calcium carbonate in two variants, together or separately. It was hypothesized that an increase in the efficiency of the ion exchange process can be obtained by binding protons—released into the solution during the ion exchange process—with OH^−^ anions released into the solution as a result of calcium carbonate dissolution and subsequent hydrolysis reaction, which in turn can improve the efficiency and effectiveness of metal removal.

## Materials and methods

Several types of biosorbents (BS) were used in preliminary studies: crushed spelt or buckwheat grains, bran of: spelt, buckwheat, rice, wheat, and brewer's spent grain (BSG) as well as, for comparison, green parts of *Pleurozium schreberii* mosses. BS rinsed with demineralized water and dried at 323 K to a constant mass were used for the study. One batch of BS prepared and homogenized in this way was stored in tightly closed PE vessels, in quantities enabling all tests, including repetitions. Tests were also carried out on protonated brewer's spent grain (P-BSG). For this purpose, BSG was conditioned for 60 min in HCl solution (0.03 mol L^−1^)^45^. After conditioning, BSG was repeatedly rinsed with demineralized water until a stable pH of water was obtained in which it was immersed and then dried for about 24 h at 323 K. Conditioning in HCl solution is the stage of BSG regeneration for reuse in the sorption process^38^. The interpretation of the results of the completed experiments is more a qualitative description of phenomena due to the fact that the kinetic and equilibrium parameters depend on the mutual proportions of the concentration and the volume of the solution, the weight of the biosorbent used, and the mass contribution of calcium carbonate used for the experiments, which was not considered in detail. Moreover, the description of the phenomena concerns a group of selected biosorbents for which detailed quantitative interpretation requires consideration of each of them separately, which may be the subject of further studies.

### Method of testing

Studies of processes for removing metal (Cu, Zn, and Pb) cations were carried out in two variants: *together* (metal salt solution (400 mL) − 2 g BS (0.5 g moss) and 0.1 g CaCO_3_), and *separately* (metal salt solution (200 mL) − 2 g BS (0.5 g mosses)) and a metal salt solution (200 mL) − 0.1 g CaCO_3_. The proportion of the volume of the solutions resulted from maintaining the same initial number of moles of metal cations (*n*_M,0_) in both variants. The solutions used in the experiments contained single metal cations or a mixture of these. The study was conducted in a static and flow-through system. In the static system, the BS and solution were placed in a beaker. The removal process was carried out with intensive stirring using a magnetic stirrer. The changes of metal concentrations were determined in the solution before (*c*_M,0_) and after (*c*_M,1_) the removal process. The reduction in the mass of mosses used in the experiments results from the fact that mosses have much better sorption properties compared with other BS. In the variants, together and separately for a 2 g sample of the mosses, the metal concentrations after the sorption process were below the limit of quantification of the method. The mosses were used for comparative purposes. Using a scanning electron microscope, an analysis was also carried out to show the differences in the surface porosity of the examined biosorbents, affecting the sorption properties in the surface layer. The sorption solutions were filtered through 0.45 μm membrane filters (Whatman). Possible loss of metal mass resulting from sorption on the filters was not greater than the determined uncertainty value of the measurement method. The studies were carried out at different time intervals, with intensive mixing by means of a magnetic stirrer.

**Figure 1:**
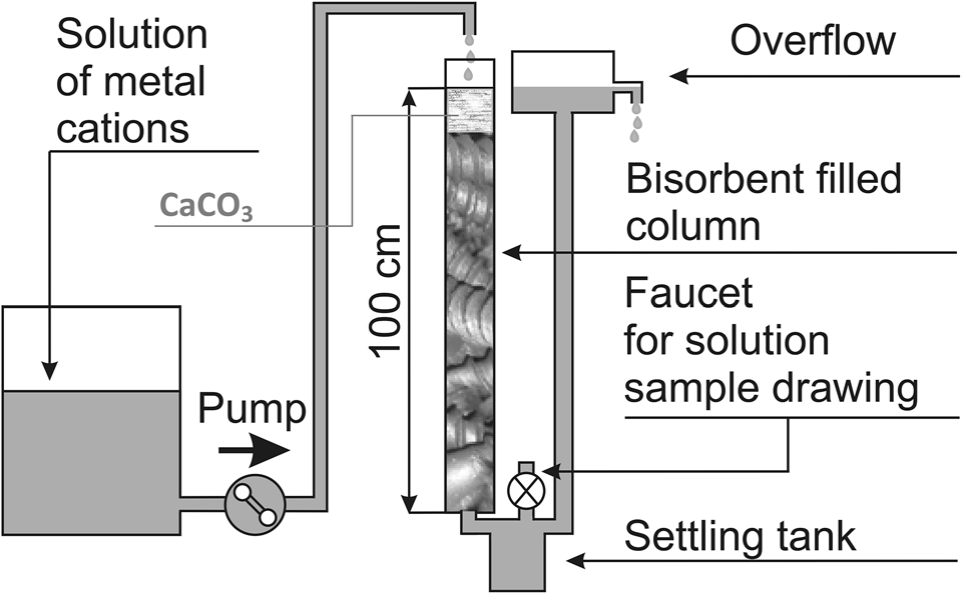
Diagram showing the sorption assemblie for a flow-through system.

Sorption processes in the flow system were studied using a polycarbonate column 100 cm long and 2 cm in diameter. In turn, the column was filled with: (1) 30 g of P-BSG, (2) P-BSG which was then introduced into the upper layer 2 g of powdered CaCO_3_, and (3) 2 g of powdered CaCO_3_ placed between cellulose layers in the upper layer of the column filled with inert glass balls similar in size to those of the BSG (diameter = 3 mm). Metal solutions were fed from the top in the amount of 200 mL h^−1^. Due to the use of a siphon, the whole volume of the column was in contact with the solution. A diagram of the set-up is shown in Fig. 1.

The experiment in the flow system was carried out using the solutions of each of the metal cations separately and the solution of the metal cation mixture.

The metal cation solutions of comparable molar concentrations were prepared for testing the removal efficiency from single-component solutions: *c*_Cu(0)_ = (0.144–0.147 mmol L^−1^, mean value 0.146 mmol L^−1^), *c*_Zn(0)_ = (0.150–0.153 mmol L^−1^, mean value 0.152 mmol L^−1^), *c*_Pb(0)_ = (0.145–0.154 mmol L^−1^, mean value 0.150 mmol L^−1^). The concentration of the sum of metal cations in the multi-component solution was 0.158–0.178 mmol L^−1^, and respectively: *c*_Cu(0)_ = (0.050–0.059 mmol L^−1^, mean value 0.054 mmol L^−1^), *c*_Zn(0)_ = (0.056–0.062 mmol L^−1^, mean value 0.059 mmol L^−1^), *c*_Pb(0)_ = (0.052–0.057 mmol L^−1^, mean value 0.055 mmol L^−1^). The solutions were passed through the column for 150 h, and samples for testing were taken periodically (Fig. 1).

The charts show the removal efficiency in the following form:

 − for the static method:
  1 $${\text{Removal}}_{S} \left( \% \right) \, = { 1}00 \, \cdot \, \left( {n_{{{\text{M}},0}} {-}n_{{{\text{M}},{1}}} } \right) \, /n_{{{\text{M}},0}}$$
 − for the flow-through method:
  2 $${\text{Removal}}_{F} \left( \% \right) \, = { 1}00 \, \cdot \, \left( {c_{{{\text{M}},0}} {-}c_{{{\text{M}},{1}}} } \right) \, /c_{{{\text{M}},0}}$$
  where: *c*_M,0_ and *c*_M,1_ are the metal concentrations in the solution before and after the removal process, *n*_M,0_ and *n*_M,1_ are the numbers of metal cations in the solution before and after the removal process; *n*_M_ = *c*_M_/*V*, where: *V* is the volume of the solution used for the experiments (L).

Changes of the concentration in the flow-through system are also interpreted as a function of the contact time of the solution with the bed.

The removal kinetics in the static method, in the *together* and *separately* systems, is described by comparing the removal efficiency (Dependence 1) at one-hour intervals for the duration of the experiment, and by comparing the kinetic coefficients determined from the pseudo-second-order reaction model^46^.

3 $$t/q_{{{\text{M}},{\text{t}}}} = { 1}/k^{\prime\prime}\left( {q_{{{\text{M}},{1}}} } \right)^{{2}} + { 1}/q_{{{\text{M}},{1}}} \cdot t$$

Under experimental conditions, *q*_M,t_ indicates the number of moles of metal removed after *t* due to the sorption on 2 g of the biosorbent and to the reaction with 0.1 g of CaCO_3_. Therefore, *q*_M,t_ for the variant *together* was calculated from the relation: (*c*_M,0_–*c*_M,t_) × 0.4, whereas for the variant *separately*: ((*c*_M,0_–*c*_M,t_) × 0.2)_BS_ + ((*c*_M,0_–*c*_M,t_) × 0.2)_CaCO3_. The subscripts BS i CaCO_3_ indicate, respectively, the removal efficiency of metal cations after *t* by the biosorbent immersed in 200 mL of the solution, and the removal efficiency of metal cations after *t* by CaCO_3_, placed in 200 mL of the solution. In this case, the reaction rate constant *kʺ* is: (0.1 g CaCO_3_ + 2 g BS)/(mmol h).

The next stage of research concerned the determination of mobile hydrogen cations bound in active biosorbent centres, and how they affect the removal process of the studied metals from solutions. For this purpose, the pH changes were determined in 200 mL of demineralized water (in which 2 g of biosorbent was immersed) titrated with a NaOH solution (c = 0.001 mol L^−1^). Before the experiment, biosorbents were rinsed with demineralized water to obtain a stable pH (5.0–5.3).

### Apparatus and reagents

The atomic absorption spectrometer (AAS) iCE 3500 from *Thermo Electron* Corporation, USA, was used to determine concentrations of heavy metals. The pH values of the solutions were determined using a CP551 pehameter, produced by Elmetron (unlimited partnership) from Zabrze (Poland), of which the absolute error of indication was: ∆pH = 0.02. The following MERCK reagents (salts of the analysed metals) were used in the study: CuSO_4_ · 5H_2_O, ZnCl_2_ and PbCl_2_, as well as CaCO_3_, HCl and NaOH. Conductivity of the water used for the studies did not exceed 1.0 µS cm^−1^. The morphological features of the samples were evaluated using a Hitachi model TM3000 scanning electron microscope (SEM). The samples were fixed on an aluminium sample stub and coated with palladium by conventional sputtering techniques (to provide their conductivity). The SEM operating voltage was 15 kV and the images were taken at the magnification of 1000 ×.

### Quality assurance and quality control

The tests were carried out in 5 repetitions. The standard deviation from the mean value determined for Cu did not exceed 10%, and 14% for Zn and Pb, respectively. Detailed data concerning the apparatus, together with the assessment and quality assurance, were published^47^.

## Results and discussion

The studies were conducted to demonstrate a synergistic effect between biosorbent and calcium carbonate in the process of removing heavy metals from the solution. According to the thesis, experiments were conducted to evaluate the impact of this effect by comparing the removal efficiency of BS, CaCO_3_ and BS + CaCO_3_, and an attempt was made to explain this phenomenon. The next stages of the research were aimed at:

 − comparing the kinetics of the removal processes of the studied metals in a static system,
 − comparing the efficiency of removing heavy metals in a static system, using different biosorbents and different initial concentrations of the tested metals,
 − indicating the occurrence of a synergistic effect of interaction in a flow-through system,
 − demonstrating that an important factor creating a synergistic effect is the binding of protons released from BS by ion exchange, by OH anions − formed by hydrolysis.

The results of the study are posted in: Supplementary information—Role of calcium carbonate in the process of heavy metal biosorption from solutions: synergy of metal removal mechanisms, Mendeley Data, V1, 10.17632/sftmm44hj6.1.

### Kinetics of the removal process

**Figure 2:**
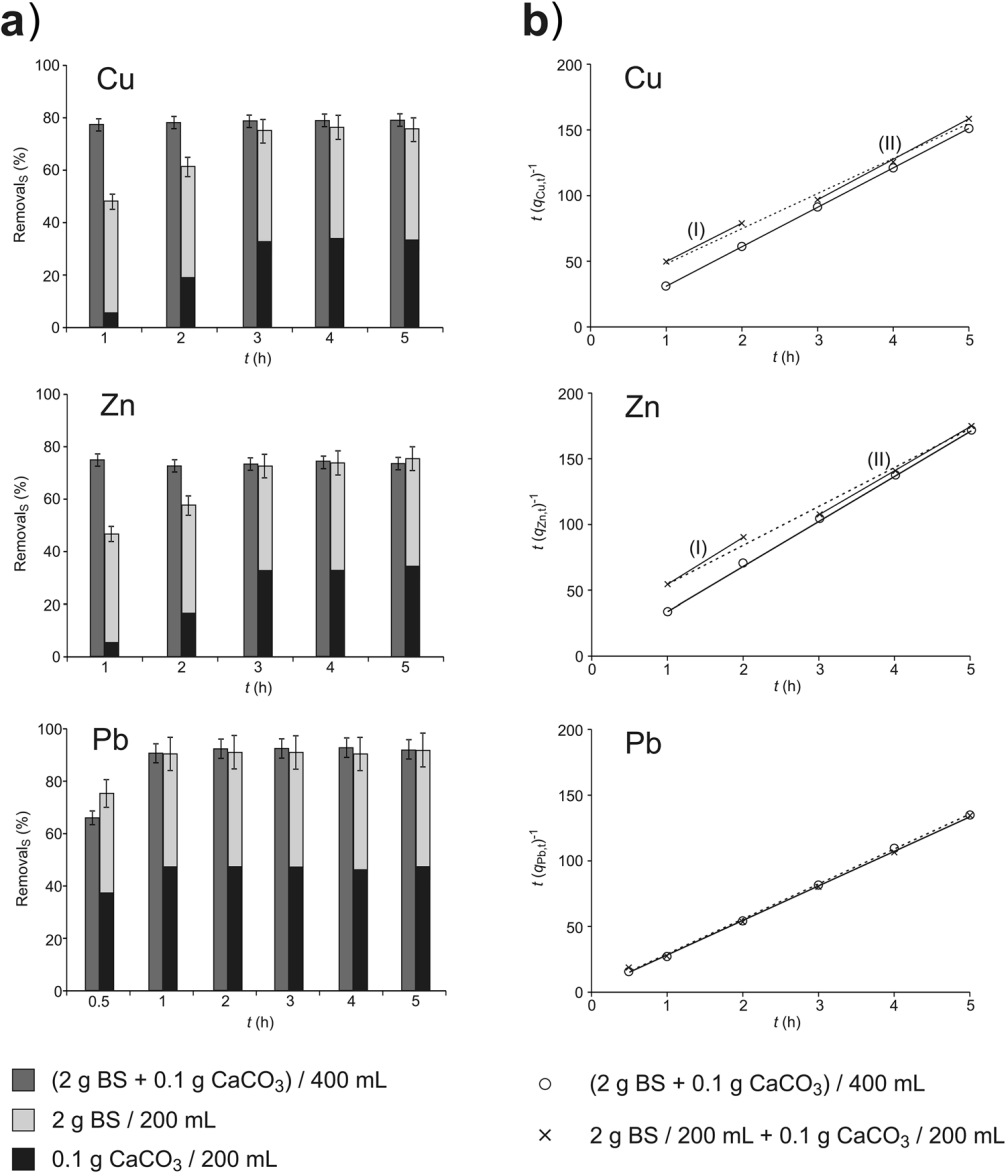
Kinetics of removal of metal cation using spelt husks and CaCO_3_ in variants: *together* gray, and *separately* light gray, dark gray, described by: (**a**) removal efficiency depending on the contact time, (**b**) pseudo-second-order reaction model.

In order to assess the temporal changes of metal concentrations in solutions, occurring during the metal removal process using BS and CaCO_3_, the removal efficiency (Dependence 1) was compared at different contact times in variants: together and separately (Fig. 2a). The pseudo-second-order reaction model (Dependence 3) was also used to interpret the results. The initial concentrations of metal solutions were: *c*_Cu,0_ = 0.104 mmol L^−1^, *c*_Zn,0_ = 0.098 mmol L^−1^, and *c*_Pb,0_ = 0.101 mmol L^−1^. The studies were conducted using spelt husks. The results are presented in Fig. 2. (All data is provided in Supplementary Table S1). The error bars indicating the uncertainty of measurement expressed by the standard deviation from the series of repetitions are marked (in the variant the composite uncertainty was determined separately).

The results presented in the graphs (Fig. 2a) show some interesting differences depending on whether the experiment was conducted *together* or *separately*. In the variant *together*, in the case of Cu and Zn, the effect of process optimization was obtained after just one hour (removal efficiency of approx. 80%). In the variant *separately*, this effect was obtained only after about 3 h. As can be seen from the graphs, this is mainly influenced by metal removal due to the reaction with CaCO_3_. In the case of contact of the spelt husk with the solution, the process stabilizes during the first hour. Other authors also note that a state close to equilibrium in the process of heterophasic ion exchange BS—solution was achieved after about 1 h of contact time^34–37^, and this is confirmed by results of our own research^38^. The lead removal process is different. After the first hour of the process, no differences in lead removal efficiency were found in both variants of the experiment. The exemplary results obtained after 30 min of contact indicate that at this time, in both variants, a state close to equilibrium was not reached.

To sum up, the principal process of metal cation removal is the ion exchange that occurs according to Dependence 4 (Ct^*z*+^ = H^+^). In the case of Cu^2+^ i Zn^2+^, in the together variant, the removal of protons desorbed from BSs according to Dependence 8 shifts the equilibrium to the right, consequently accelerating the ion exchange process, while simultaneously increasing the number of active centres available for Cu^2+^ and Zn^2+^. In the separately variant, the equilibrium of the ion exchange process also occurs during the first hour, but with lower efficiency as regards Cu^2+^ and Zn^2+^, according to Dependence 4. The diagrams demonstrate that the slowest process are the reactions occurring between CaCO_3_ and the metal cation (the separately variant), resulting in the precipitation of poorly soluble compounds (Dependences 5 and 7) where, in the case of Cu^2+^ and Zn^2+^, the equilibrium is reached after about 3 h.

The application of the pseudo-second-order reaction model (Fig. 2b) to compare the two variants is poorly justified. Under experimental conditions of the *together* variant, the value *q*_M,t_ for Zn and Cu is constant, which is demonstrated by the removal efficiency shown in Fig. 2a. Therefore, in the case of the linear function (Dependence 3), the variable y = *t*/q_M,t_ depends only on *t*. The interpretation of the *separately* variant for Zn and Cu, using the pseudo-second-order reaction model, reveals some differences in the first stage of the process (marked as I in Fig. 2b), compared to a further stage of the process (stage II). This effect (more noticeable in Fig. 2a) results from the fact that, in the *separately* variant, the BS/solution equilibrium is already reached in the first hour of the experiment, and the reaction of metals with CaCO_3_ is responsible for the changes. During the third and the following hours in the *separately* variant, the equilibrium is also reached in the CaCO_3_/solution system. This effect is not observed in the case of the faster lead removal process.

One reason for the lack of the synergistic effect in the case of Pb^2+^ cations is probably the high affinity of this cation for the active centres of BSs. The authors indicate that the Pb^2+^ affinity is highest among the metal cations analysed in this article^39^. Furthermore, as demonstrated in a few articles involving protons, the affinity of Pb^2+^ cations is significantly higher than that of H^+^: H^+^  < Zn^2+^  < Ni^2+^  < Cu^2+^  < Cd^2+^  < Pb^2+^^40^. Therefore, the removal of H^+^ cations from the solution due to the reaction with CaCO_3_ does not significantly increase the Pb^2+^ biosorption.

The pseudo-second-order reaction model is often used to describe the kinetics of biosorption^41^, but in the analysed case, a better visualization of the synergistic effect is achieved by comparing the removal efficiency (Fig. 2a).

At a later stage of the experiment it was checked whether similar effects are observed for other biosorbents. For this purpose, the removal efficiency after 1 h of contact was compared.

### Efficiency of metal removal depending on its initial concentration in the solution

**Figure 3:**
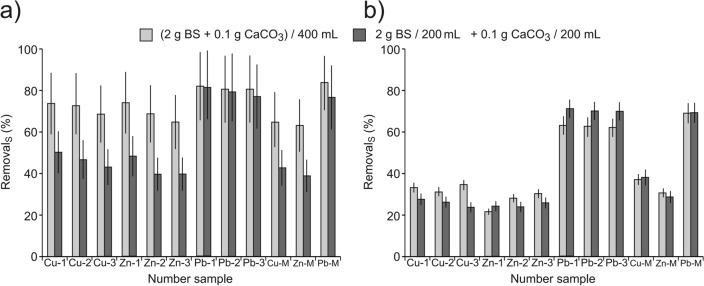
Efficiency of removal metal cations with the use of BS and CaCO_3_, in variants: *together* light gray and *separately* dark gray; (**a**) average values for bran from spelt, buckwheat, rice and wheat as well as for BSG, (**b**) for crushed spelt and buckwheat grains.

Table 1 presents collected data on the initial concentrations of metals in the solutions used for their removal with BS and CaCO_3_ in variants: together and separately. Table 1 contains the same symbols, with the subscript "0", as used in Fig. 3. The detailed study results and the conversion method are presented in the Supplementary Materials.

**Table 1:** The initial concentrations of metals *c*_M(0)_ (mmol L^−1^) in solutions used for their removal.

Single-metal cation solutions									Mixture of metal cations		
Cu			Zn			Pb			Cu	Zn	Pb
Cu-1_0_	Cu-2_0_	Cu-3_0_	Zn-1_0_	Zn-2_0_	Zn-3_0_	Pb-1_0_	Pb-2_0_	Pb-3_0_	Cu-M_0_	Zn-M_0_	Pb-M_0_
0.076	0.110	0.158	0.064	0.108	0.155	0.072	0.098	0.160	0.054	0.048	0.062

The effects of removal after 1 h of contact time, obtained in two variants: *together* ((2 g BS + 0.1 g CaCO_3_)/400 mL of solution) and *separately* (2 g BS/200 mL of solution + 0.1 g CaCO_3_/200 mL of solution), are presented in Fig. 3. (All data is provided in Supplementary Table S2).

Biosorbents were grouped taking into account comparable sorption properties. Figure 3a represents the average values of the removal efficiency by spelt-, buckwheat-, rice- and wheat bran and BSG. In Fig. 3b, the crushed grain of spelt and buckwheat was grouped. The figures show mean values of standard deviation indicating not the measurement uncertainty but differences in removal efficiency of each of the grouped BS.

The presented results indicate a clear synergistic effect of BSG and CaCO_3_ activity during copper and zinc removal using various types of bran and brewer’s spent grain (Fig. 3a), also for removal of these metals from the multi-component solution. This effect is not observed in the case of lead removal and in the variant presented in Fig. 3b. As mentioned earlier, the uncertainty from 5 repetitions, expressed by standard deviation from the mean value determined for Cu, did not exceed 10% and 14% for Zn and Pb, respectively.

**Figure 4:**
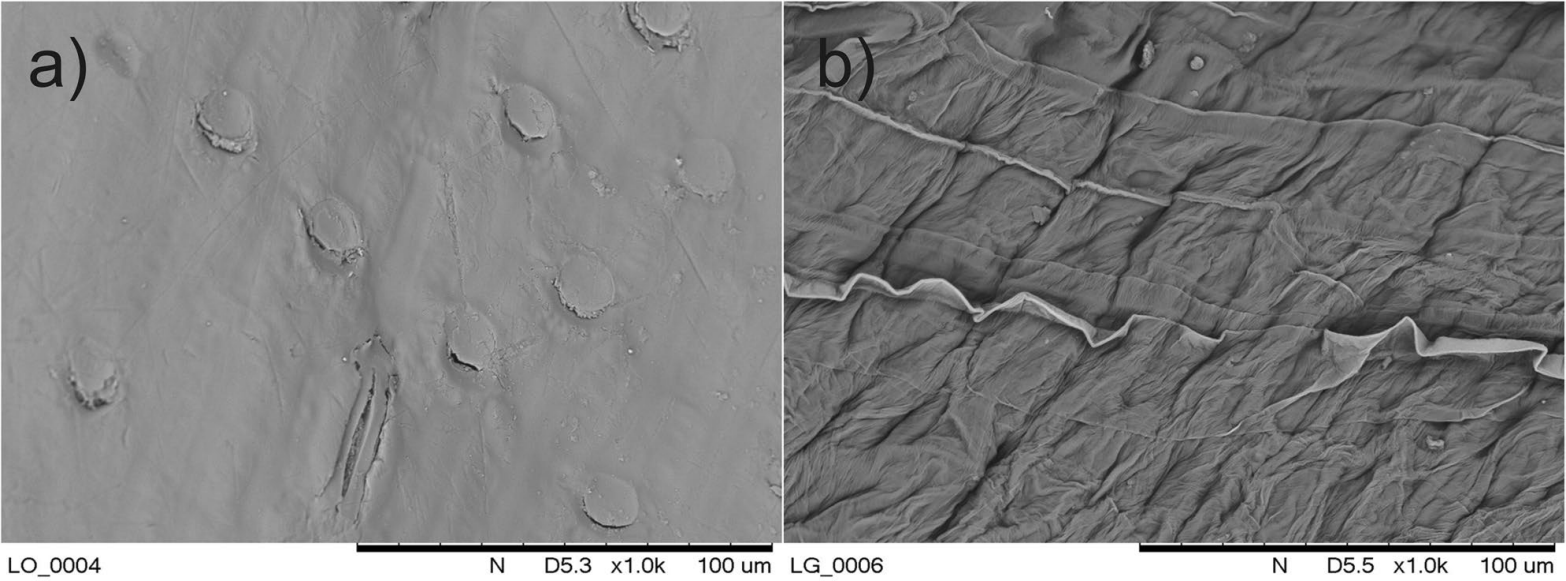
Surface structure of spelt grains (**a**) and bran (**b**).

The presented results also indicate the weaker sorption properties of crushed spelt and buckwheat grains in comparison with bran and BSG, as well as a high affinity of lead to active BS centres. This is also confirmed by other studies^38^. As mentioned above, the sorption of metal cations is the process of heterophasic ion exchange occurring mainly on the surface of biosorbents and involves functional groups capable of binding cations from the solution—for example, carboxyl (–COOH), amine (–NH_2_) and hydroxyl (–OH)^42,43^. Therefore, the sorption efficiency results, among other things, from the surface porosity and the number of functional groups per surface unit. Figure 4 compares the surface structure of spelt grains and bran.

As shown in the photographs presented in Fig. 4, the development of the BS surface improves the sorption properties.

**Figure 5:**
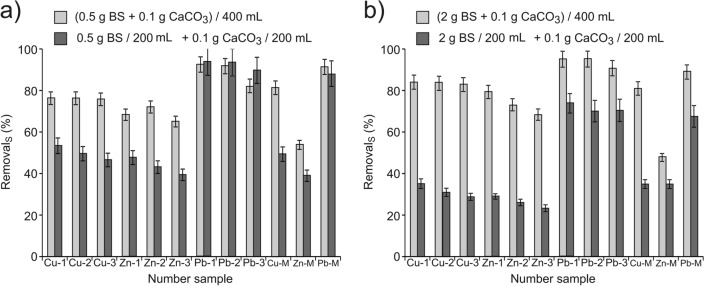
Efficiency of metal cation removal with the use of BS and CaCO_3_, in variants: *together* light gray and *separately* dark gray; (**a**) using *Pleurozium Schreberii* mosses (**b**) using P-BSG.

Figure 5 presents the results regarding the efficiency of removal of the studied metals using *Pleurozium schreberii* mosses (Fig. 5a) and P-BSG (Fig. 5b). Moss and P-BSG are characterized by much better sorption properties. Only 0.5 g of moss was used during each experiment. The error bars determine the measurement uncertainty for the variant *together* and the composite uncertainty for the variant *separately*.

The data presented in Fig. 5 indicate that, of the analysed BSs, it is the moss (without going into the validity of this thesis, as it is not the subject of the analysis) that has the best developed BS/solution contact surface (a quarter of the moss mass, compared with other BSs, was used for the experiments). Therefore, the removal effects per unit mass are the best in this case. However, the results presented in Fig. 5 also indicate a clear synergistic effect of BS and CaCO_3_ in the case of copper and zinc removal and, in the case of P-BSG, also for lead removal. Therefore, it can be presumed that, according to the hypothesis, the reaction occurring according to Eq. (8) affects the activation of active BS centres for binding metal cations from the solution, and the synergy effects increase with an increased number of protons bound in the active BS centres. In order to confirm this thesis, it is important to compare the data presented in Figs. 3a and 5b for brewer’s spent grain (BSG) and P-BSG. Better removal effects and a more significant synergistic effect are observed when P-BSG is applied. Better metal removal efficiency of P-BSG compared with BSG has been confirmed before^38^.

**Figure 6:**
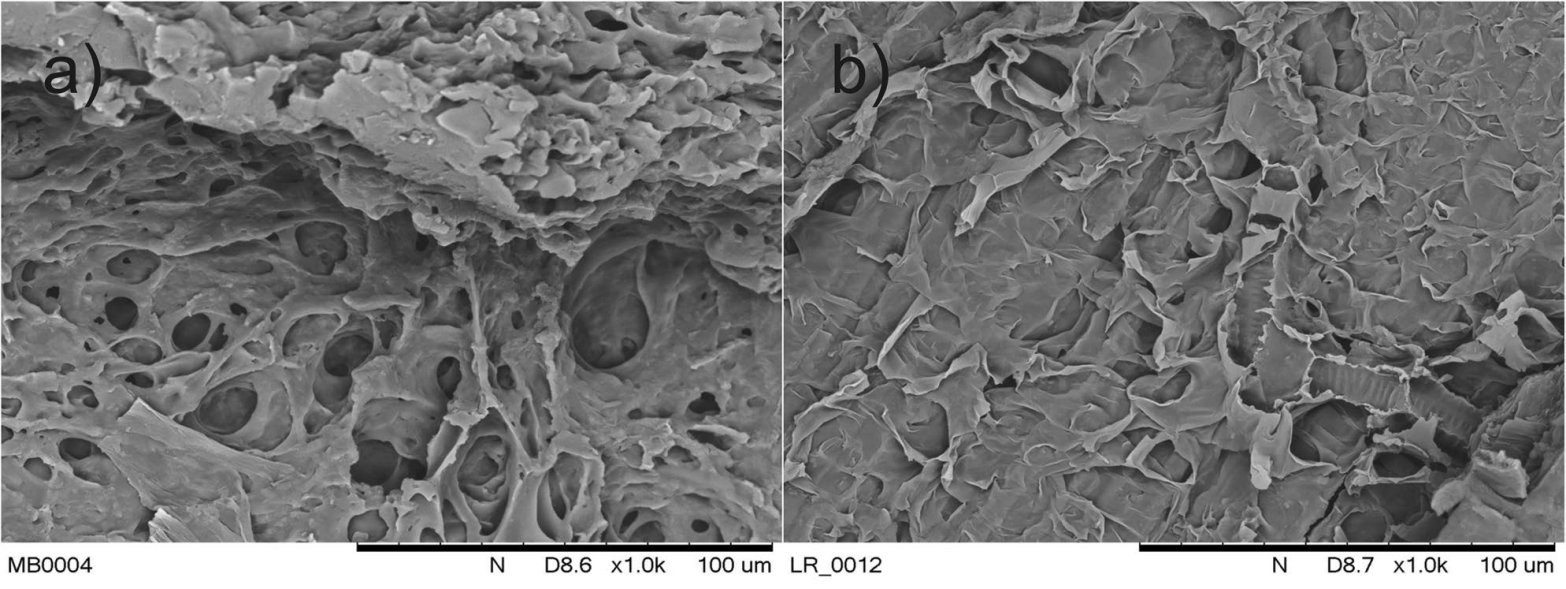
Surface structure of BSG (**a**) and P-BSG (**b**).

Figure 6 shows photographs of the surface structure of BSG and P-BSG.

Unlike in the case of spelt grains and bran (Fig. 4), the structure of BSG, characterized by poorer metal cation sorption properties, appears to be more developed compared with P‑BSG, which might results from the action of HCl solution during protonation. This indirectly supports the thesis that synergistic effects increase with the increased number of protons bound in active BS centres. It is reasonable to assume that the surface porosity, namely the BS/solution contact surface, primarily affects the sorption efficiency rather than the synergistic effect.

In order to further evaluate the synergy effects, changes in the concentration of sorbed metal cations in the flow system were studied.

### Removal of Cu^2+^, Zn^2+^ and Pb^2+^ cations from solutions in the flow system

**Figure 7:**
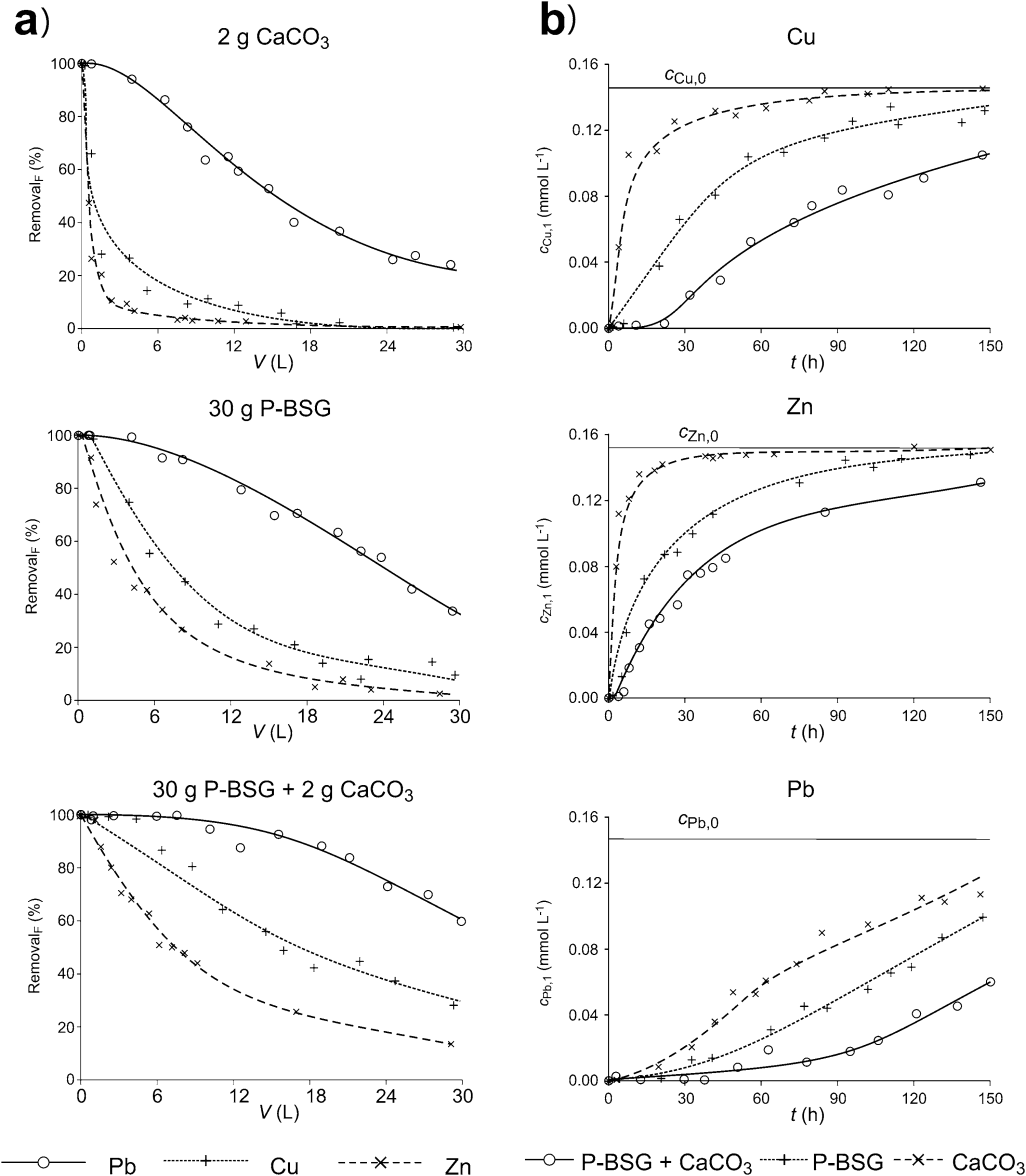
Comparison of the removal efficiency of Cu^2+^, Zn^2+^ and Pb^2+^ cations from single-component solutions in the column filled with P-BSG only, P-BSG with CaCO_3_ addition or by inert filling with addition of CaCO_3_, in functions: Removal _*F*_ = ƒ(*V*) (**a**) and *c*_M,1_ = ƒ(*t*) (**b**). The standard deviation from the mean value determined for Cu did not exceed 10%, and 14% for Zn and Pb, respectively.

As mentioned, the experiments were carried out using single-component solutions and a metal cation mixture solution. The metal removal efficiency (Dependence 2) from single-component solutions depending on the volume of solution passed through the column: The removal _*F*_ = ƒ(*V*) and in the function of the time variation of metal concentrations in the solution after passing through the column: *c*_M,1_ = ƒ(*t*) is shown in Fig. 7. (All data is provided in Supplementary Table S3). P-BSG was used for the experiments.

Comparison of the position of the curves presented in the graphs indicates the affinity of the studied metals cations to P-BSG: Pb^2+^ > Cu^2+^ > Zn^2+^ and the efficiency of metal cation removal due to reaction with calcium carbonate. In this case, the better removal efficiency of Pb cation compared to Cu and Zn cations is probably due to the fact that the solubility product of PbCO_3_ is 2 orders smaller than for ZnCO_3_ and 3 orders smaller than for CuCO_3_. A synergy effect is also visible, particularly in the case of Cu and Zn cation removal. As in the case of static experiments (Fig. 3b), the removal efficiency of these metals by a mixture of P-BSG and CaCO_3_ is greater than the sum of removal efficiency by P-BSG and CaCO_3_ separately. For example, under experimental conditions, after passing 6 L of copper solution through a column filled with P-BSG and CaCO_3_, the removal efficiency is close to 100%, whereas when passing the same volume of solution of P-BSG and CaCO_3_ separately, the removal efficiency is about 55% and 15% respectively.

**Figure 8:**
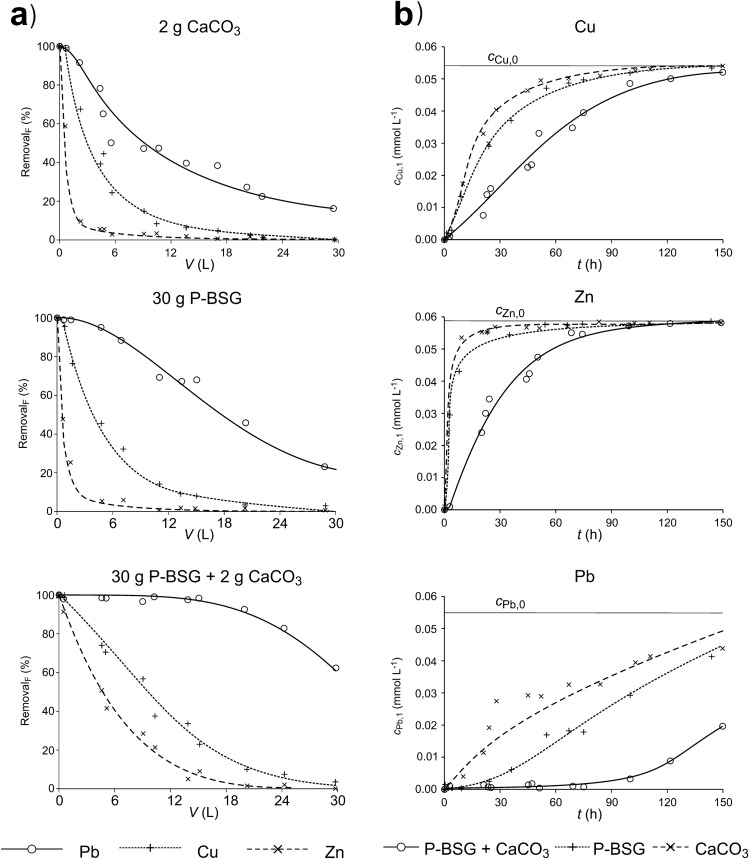
Comparison of the removal efficiency of Cu^2+^, Zn^2+^ and Pb^2+^ cations from the multi-component solution in the column filled with P-BSG, P-BSG with CaCO_3_ addition and with inert filling with addition of CaCO_3_. The standard deviation from the mean value determined for Cu did not exceed 10%, and 14% for Zn and Pb, respectively.

The next stage of the study concerned the assessment of the removal efficiency of Cu, Zn and Pb cations from a multi-component solution. For the study, as mentioned above, a solution with a comparable initial metal cation concentration was used. The sum of concentrations was within the range: 0.95–1.09 mmol L^−1^ and was comparable to the initial metal concentrations in the solutions used in the previous experiment: 0.94–1.11 mmol L^−1^. The results of the experiment are presented in Fig. 8. The test results presented in Fig. 8 confirm the synergy effects, this time particularly with regard to lead cations. The efficiency of lead cation removal by the mixture of P-BSG and CaCO_3_ with effectiveness not less than 90% was still observed when about 20 L of solution was passed through the column, and this indicates the removal of about 6 mmol of lead cations. When P-BSG is used alone, the removal efficiency after passing the 20 L solution through the column was about 40% and about 25% in the case of CaCO_3._ Physical adsorption of carbonates and hydroxides of the tested metals—precipitated as a result of chemical reactions—on the BS surface, may have some significance in the removal process.

In order to confirm that the concentration of protons bound in BS functional groups affects the synergistic effect, a titrimetric analysis of the BSs used in the study was carried out.

### Interpretation of chemical reactions occurring during the removal process

The main mechanism of sorption of the examined heavy metals (M^2+^: Cu^2+^, Zn^2+^, Pb^2+^) in biosorbents, which is also confirmed by other authors^48^, is a ion exchange according to the scheme:

4 $${\text{2CtR}}_{z} + z{\text{M}}^{{{2} + }}{ \leftrightarrows } {\text{2Ct}}^{z + } + z{\text{MR}}_{{2}}$$

where: Ct^*z*+^—cation originally associated with the active centers of the biosorbent, R^−^—active centers that bind cations in the biosorbent, M^2+^—cation of heavy metal sorbed, *z*—Ct valence.

However, the process of removing heavy metal cations from solutions using CaCO_3_ is complex. The basic process is the reaction of CaCO_3_ dissolution, whose equilibrium state results from a solubility product (25 °C): *K*_sp_ = 4.7 × 10^−9^^44^. The following reactions are mainly precipitation of metals in the form of poorly soluble carbonates:

5 $${\text{M}}^{{{2} + }} + {\text{ CO}}_{{3}}^{{{2} - }} { \leftrightarrows }{\text{MCO}}_{{3}}$$

and a first-degree hydrolysis reaction:

6 $${\text{CO}}_{{3}}^{{{2} - }} + {\text{ H}}_{{2}} {\text{O}}{ \leftrightarrows }{\text{HCO}}_{{3}}^{ - } + {\text{ OH}}^{ - }$$

which also results in the precipitation of sparingly soluble hydroxides of the studied metals (*K*_sp(CuCO3)_ = 2.5 × 10^−10^, *K*_sp(ZnCO3)_ = 3.0 × 10^−11^, *K*_sp(PbCO3)_ = 4.7 × 10^−13^)^44^:

7 $${\text{M}}^{{{2} + }} + {\text{ 2OH}}^{ - } { \leftrightarrows }{\text{M}}\left( {{\text{OH}}} \right)_{{2}}$$

The precipitation process for some metals can lead to the precipitation of complex compounds, e.g. basic copper carbonate or basic lead carbonate: CuCO_3_·Cu(OH)_2_, PbCO_3_·Pb(OH)_2_.

Another reaction, according to the hypothesis, is to increase the efficiency of the ion exchange process as a result of successive removal of protons bound in biosorbent active centres. Removal of protons occurs as a result of the reaction with hydroxyl anions released into the solution by hydrolysis:

8 $${\text{HR }} + {\text{ OH}}^{ - }{ \leftrightarrows } {\text{R}}^{ - } + {\text{ H}}_{{2}} {\text{O}}$$

As a result, a heterophasic equilibrium is established between Ca^2+^ cations derived from CaCO_3_ and cations of the studied metals (M^2+^: Cu^2+^, Zn^2+^, Pb^2+^).

In this case, taking into account the fact that the affinity of the cations to the active centres of most biosorbents increases in the series: Ca^2+^ < 2H^+^ < M^2+^ (Zn^2+^, Cu^2+^, Pb^2+^)^38^, the replacement of protons in the solution by calcium cations will shift the heterophasic balance towards MR_2_ formation.

### Determination of the mobile proton concentration in various biosorbents

**Figure 9:**
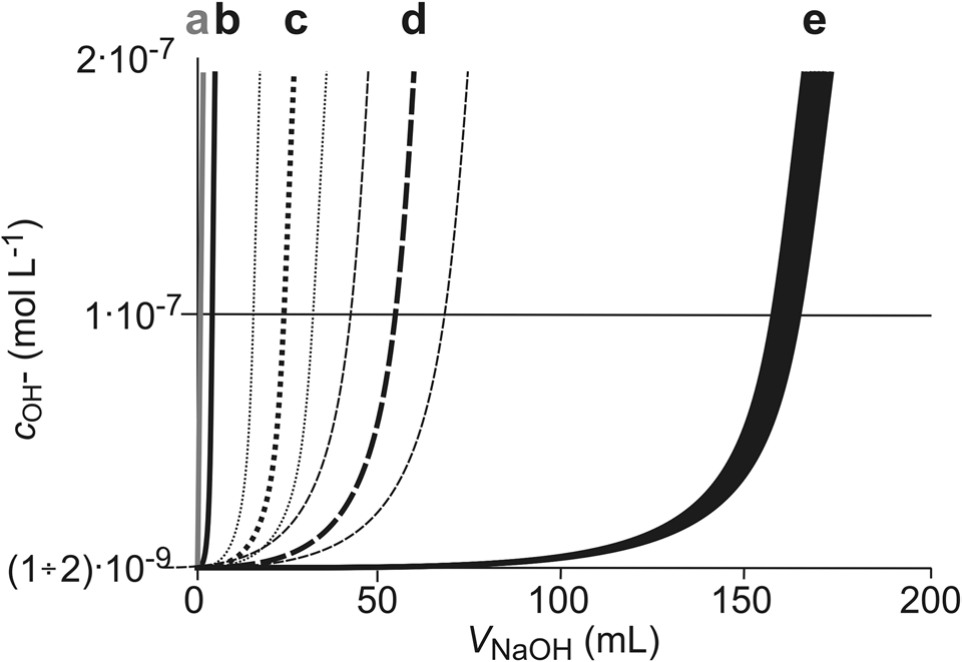
Effect of added BS on changes of OH^−^ ion concentration during titration of 200 mL deionised water with NaOH standard solution (0.001 mol L^−1^); determinations: (**a**) changes in calculated OH^−^ ion concentration values, (**b**) changes in OH^−^ ion concentration during titration of water used for experiments (pH0 = 5.17), (**c**) range of OH^−^ ion concentration changes in titrated solutions in which 2 g of crushed spelt or buckwheat grains were placed, (**d**) range of OH^−^ ion concentration changes in titrated solutions in which 2 g of bran: spelt, buckwheat, rice, wheat or BSG were placed, (**e**) range of OH^−^ ion concentration changes in titrated solutions in which 2 g P-BSG were placed.

According to the previously described experiment, the standard NaOH solution (0.001 mol L^−1^) was used for titration of an aqueous solution containing immersed BS. Figure 9 presents changes in the concentration of hydroxide ions during titration of demineralized water, in which unprotonated and protonated BS (2 g) were immersed. Concentration of hydroxide ions was calculated on the basis of pH measurements taking into account the actual volume of the solution: *V* (mL) = 200 + *V*_NaOH_, where *V*_NaOH_—the volume of the added titrant. In the graph, for comparison, the following were recorded: a—changes of theoretical OH^−^ ion concentration values calculated according to the ionic product of water and the pH definition, (pH_0_ = 5.15), b—changes in OH^−^ ions concentration during titration of water used for experiments (pH_0_ = 5.17), c—range of changes in OH^−^ ions concentration in titrated solutions in which 2 g of crushed spelt or buckwheat grains were placed, d—range of OH^−^ ions concentration changes in titrated solutions in which 2 g of spelt-, buckwheat-, rice-, wheat bran or BSG were placed, e—range of OH^−^ ions concentration changes in titrated solutions in which 2 g P-BSG were placed.

The results presented in the graph show that in the case of P-BSG (curve e), about 160 mL of NaOH solution (c_NaOH_ = 0.001 mol L^−1^) was used to neutralize the solution in which 2 g of BSG were immersed, which means that—according to Dependence 8, in conversion to 1 g P-BSG—0.08 mmol of protons were released into the solution. Non-acidified BSG and bran released about 0.025 mmol of protons (curve d), while for grains of spelt and buckwheat this value was about 0.01 mmol (curve c). What is important here is the number of active centers binding mobile cations, which depends on the development of the surface (Figs. 4 and 6) and the share of protons in the total number of cations bound.

In this respect, the tested BS can be divided into three groups differing in the number of released protons involved in the neutralization of OH^−^ ions introduced into the solution during the titration process: (1) crushed grains of spelt and buckwheat, (2) bran of different cereals and BSG and (3) P-BSG. It should be noted that such a division occurs when studying the synergy effects presented in Figs. 3 and 5b i.e. that the synergy effect increases as the number of protons participating in the ion exchange process increases. The reason for the lack of synergy effect in the case of sorption of Pb^2+^ cations remains undetermined, in contrast to the clearly visible effect for Cu^2+^ and Zn^2+^ after the first hour of the experiment (Figs. 2 and 3). It can be assumed that this results from the solubility products of the metal carbonates participating in the reaction: *K*_sp(CaCO3)_ = 4.7 × 10^−9^, *K*_sp(CuCO3)_ = 2.5 × 10^−10^, *K*_sp(ZnCO3)_ = 3.0 × 10^−11^, *K*_sp(PbCO3)_ = 4.7 × 10^−13^^44^. As the presented figures show, the solubility product of lead carbonate is at least 2 orders smaller than the others, which significantly reduces the concentration of CO_3_^2−^ in the solution (Dependence 5) and, consequently, reduces the concentration of OH^−^ ions generated by hydrolysis (Dependence 6).

Summarizing the results of the study, it can be concluded that the removal of metal cations by BS occurs mainly by ion exchange (Dependence 2). In this case, a heterophasic equilibrium is established between the concentration of heavy metal cations sorbed from solution and other cations, including protons, originally bound to the BS.

The use of CaCO_3_ in the removal process results in the precipitation of poorly soluble carbonates and heavy metal hydroxides (Dependence 3 and 5).

In the combined system (BS + CaCO_3_) all the reactions mentioned above take place, but in addition there is proton binding by hydroxide anions released due to hydrolysis (Dependence 6). The benefits of this are a shift in heterophasic ion exchange toward binding of heavy metal cations in BS (Dependence 2) and, due to binding of hydroxyl anions, a shift in hydrolysis equilibrium.

## Conclusions

The presented research results indicate that supporting biosorption processes using calcium carbonate addition measurably improves the efficiency of removal of heavy metal cations from aqueous solutions. This effect results from the binding of protons, released into the solution during the ion exchange process, by OH-anions released into the solution due to dissolution of calcium carbonate and the subsequent hydrolysis reaction. This is particularly important in the case of multiple-bed technology by regeneration with acid solutions. As shown by the example of P-BSG, the synergy effects are then best seen. It has been shown that:

 − in a static system, the removal efficiency for Cu and Zn increases from 20 to 50% depending on the BS used,
 − in a static system, the measurable effect of improving lead removal efficiency occurs only with biosorbents whose active centers are saturated with protons,
 − in a flow-through system, under experimental conditions using P-BSG, it was demonstrated that the addition of powdered CaCO_3_ in a weight ratio of 1 g CaCO_3_: 15 g P-BSG increases the removal efficiency of all metals tested by 20–30%.

## Supplementary Information

Supplementary Table S1.

Supplementary Table S2.

Supplementary Table S3.

## Data Availability

Supplementary information—Role of calcium carbonate in the process of heavy metal biosorption from solutions: synergy of metal removal mechanisms, Mendeley Data, V1, 10.17632/sftmm44hj6.1.
